# The Effects of Allicin, a Reactive Sulfur Species from Garlic, on a Selection of Mammalian Cell Lines

**DOI:** 10.3390/antiox6010001

**Published:** 2016-12-26

**Authors:** Martin C. H. Gruhlke, Carole Nicco, Frederic Batteux, Alan J. Slusarenko

**Affiliations:** 1 Department of Plant Physiology, RWTH Aachen University, Worringer Weg 1, Aachen 52074, Germany; alan.slusarenko@bio3.rwth-aachen.de; 2 Laboratoire d’Immunologie biologique, Hôpital Cochin, Paris 75679, France; carole.nicco@gmail.com (C.N.); frederic.batteux@cch.aphp.fr (F.B.)

**Keywords:** garlic, *Allium sativum*, allicin, tumour cell lines, glutathione, redox

## Abstract

Garlic (*Allium sativum* L.) has been used as a spice and medicinal plant since ancient times. Garlic produces the thiol-reactive defence substance, allicin, upon wounding. The effects of allicin on human lung epithelium carcinoma (A549), mouse fibroblast (3T3), human umbilical vein endothelial cell (HUVEC), human colon carcinoma (HT29) and human breast cancer (MCF7) cell lines were tested. To estimate toxic effects of allicin, we used a standard MTT-test (methylthiazoltetrazolium) for cell viability and ^3^H-thymidine incorporation for cell proliferation. The glutathione pool was measured using monobromobimane and the formation of reactive species was identified using 2′,7′-dichlorofluoresceine-diacetate. The YO-PRO-1 iodide staining procedure was used to estimate apoptosis. Allicin reduced cell viability and cell proliferation in a concentration dependent manner. In the bimane test, it was observed that cells treated with allicin showed reduced fluorescence, suggesting glutathione oxidation. The cell lines tested differed in sensitivity to allicin in regard to viability, cell proliferation and glutathione oxidation. The 3T3 and MCF-7 cells showed a higher proportion of apoptosis compared to the other cell types. These data show that mammalian cell lines differ in their sensitivity and responses to allicin.

## 1. Introduction

Garlic (*Allium sativum* L.) has been used as a spice and medicinal plant since ancient times; the earliest known report of garlic’s medicinal use is written in the Egyptian Codex Ebers from the 16th century B.C. [1]. Certain ingredients of garlic, in particular the thiosulfinates, have strong antimicrobial properties against a broad range of bacteria and fungi; and even a viricidal effect of garlic against different human-pathogenic viruses has been reported [2]. Furthermore, several garlic substances are physiologically active in mammals and are mainly described as health promoting, for example by reducing low-density-lipoprotein (LDL), which correlates with the development of atherosclerosis and cardiovascular diseases [3,4,5].

**Scheme 1:**
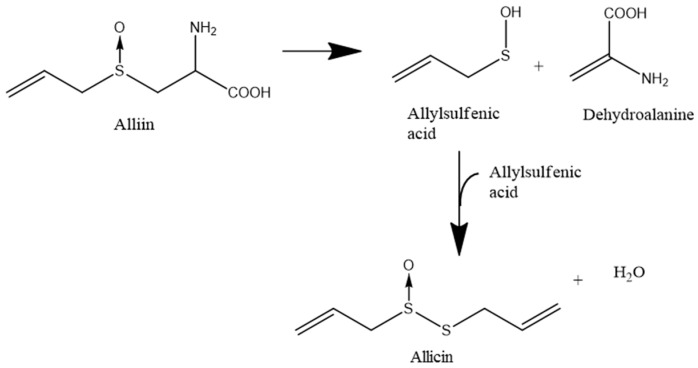
Biosynthesis of allicin. The nonproteinogenic amino acid alliin is converted by the activity of the alliinase enzyme to allyl sulfenic acid and dehydroalanine. Two molecules of allyl sulfenic acid condense spontaneously to one molecule of allicin.

The quantitatively most important compound produced by freshly damaged garlic is allicin (diallylthiosulfinate), which is formed from the non-proteinogenic amino acid alliin (allyl cysteine sulfoxide). Allicin is formed from alliin in a two-step reaction: In the first step the enzyme alliinase (a CS-lyase, E.C. 4.4.1.4) converts alliin to allylsulfenic acid and dehydroalanine. In a second step, two molecules of allylsulfenic acid condense spontaneously to one molecule of allicin (Scheme 1) [6].

Allicin’s structure and biological properties were first described by Cavallito and Bailey in 1944 [7,8]. Allicin is highly reactive with thiol-groups but under certain conditions it also reacts with itself forming further compounds which can also be bioactive, such as vinyl-dithiins, ajoene and polysulfanes [9]. Since allicin is able to oxidize biomolecules such as glutathione or protein cysteine residues under physiological conditions, it fulfils the definition of a Reactive Sulfur Species (RSS) [10]. Because thiol groups are ubiquitously present in all living cells, allicin is a dose-dependent biocide and has the capability to kill all eukaryotic cells. In 1960, it was first reported that tumour cells were killed when incubated in allicin solution [11]. Allicin is readily membrane permeable [12], and easily enters cells and reacts with cellular thiols such as glutathione [13] or cysteine residues in proteins [14]. As a consequence, enzymes with accessible reactive cysteines can be affected in their function [15]. It was suggested that the inactivation of essential enzymes might explain allicin’s toxicity. Furthermore, it has been shown that allicin is able to induce apoptosis in different cell types [16,17,18,19,20].

Although chemically allicin is an oxidizing agent and the thiosulfinate group oxidizes thiols, or more specifically thiolate ions, in nutritional physiology, allicin and garlic products in general, are regarded as having antioxidant properties [21]. Allicin certainly acts as a “physiological antioxidant” [22] by inducing the cellular “phase II detoxification system” [23]. Allicin activates the redox-dependent mammalian transcription factor Nrf-2 the localization of which depends upon the Keap1 protein (Kelch-like ECH1-associated protein) [24,25], presumably by oxidizing the regulatory cysteine-residues, as demonstrated for the YAP1-transcription factor which is a functional homologue of the Nrf-2 system in yeast. Furthermore, sulfenic acids, which are intermediates in allicin biosynthesis (see Scheme 1) and have been described as “ultimative antioxidants”, can also be released by allicin and act as traps for oxygen radicals such as peroxyl radicals [26]. 

An early observation on the effects of allicin on cancer cells was that tumour tissue taken from a mouse, and incubated in an allicin solution or saline buffer before intraperitoneal inoculation, failed to grow, in contrast to the untreated controls [11]. Furthermore, it was shown that allicin inhibited cell proliferation [27] and induced apoptosis in several cell lines via caspase-dependent and -independent pathways [16,17,24,28]. The inhibition of cell-proliferation by allicin was linked to its microtubule-disrupting properties as shown for mouse fibroblasts [18]. In addition, it was demonstrated that polysulfanes derived from allicin targeted microtubules, leading to cell-cycle arrest and finally to apoptosis [29,30]. 

Although allicin is quantitatively the most prominent thiosulfinate released by freshly damaged garlic tissue, a plethora of other compounds are produced. For example, thiosulfinates with side chains other than allyl groups are present in garlic juice as methyl- or propyl- groups, but also conjugated with amino acids [31,32,33]. Thus, whether a particular biological activity of garlic juice is due to allicin or some other component must be determined. A simple indicator for this is to demonstrate a dose response based on the allicin content of garlic juice to parallel treatments with pure allicin. Unfortunately, many studies use garlic-derived material instead of pure compounds. Therefore, in the work reported here, we tested both synthetic allicin and garlic juice, using various assays to monitor the effects on different human cell lines. Furthermore, it is important to understand the effects of allicin on mammalian cells, not only because it is a common foodstuff, but also in order to realize its full potential as a therapeutic in the treatment of infections and as an antitumour agent.

## 2. Materials and Methods

### 2.1. Cell Lines and Cultivation

Cell lines: A549 (human alveolar basal epithelial adenocarcinoma cell, ATCC#CCL-185), NIH 3T3 (neonatal fibroblasts from Mouse, ATCC#CRL-1658) HUVEC (human umbilical vein endothelial cells), HT29 (human colorectal epithelial carcinoma, ATCC#HTB-38) and MCF7 (human mammary carcinoma, ATCC#HTB-22) were from the Department of Immunology, Cochin Hospital, Paris Descartes University, Paris, France. Cells were cultivated in complete medium containing RPMI Medium (Roswell Park Memorial Institute Medium, Invitrogen) containing 10% FCS (foetal calf serum) and 1% Penicillin/Streptomycin (10,000 U/mL) (Invitrogen, Waltham, MA, USA). Cells were incubated at 37 °C and 5% CO_2_. 

### 2.2. Methylthiazoltetrazolium (MTT)-Assay

Cells were seeded at 1 × 10^4^ cells for NIH3T3 and HUVEC and 2 × 10^4^ for A549, MCF7, HT29 cells per well, in 100 µL complete medium, into 96-well plates and treated with garlic juice or allicin to obtain the final concentrations as indicated. The cells were incubated for 24 h at 37 °C and 5% CO_2_. Afterwards, 50 µL MTT-solution (methylthiazoltetrazolium, 2 mg·mL^−1^ in phosphate buffered saline) were pipetted into each well and incubated for 4 h at 37 °C. After washing cells with PBS, formazan was dissolved from the cells by adding 100 µL DMSO (dimethyl sulfoxide). Absorption was measured at 550 nm in a micro-platereader (VICTOR 2; PerkinElmer, Paris, France) [34].

### 2.3. Cell Proliferation

Cells were seeded at 1 × 10^4^ cells for NIH3T3 and HUVEC and 2 × 10^4^ for A549, MCF7, HT29 cells per well, in 100 µL complete medium, into 96-well plates and treated with garlic juice or allicin to obtain the final concentrations as indicated. The cells were incubated for 24 h at 37 °C and 5% CO_2_. ^3^H-thymidine (1 µCi·mL^−1^) was added to the culture medium six hours before the end of the incubation period. The thymidine incorporation was quantified by scintillation counting [35]. 

### 2.4. Glutathione Determination Using Monochlorobimane

Cells were seeded at 1 × 10^4^ cells for NIH3T3 and HUVEC and 2 × 10^4^ for A549, MCF7, HT29 cells per well, in 100 µL complete medium, into 96-well plates and treated with garlic juice or allicin to obtain the final concentrations as indicated. The cells were incubated for 24 h at 37 °C and 5% CO_2_. At the end of the incubation period, cells were washed twice in PBS and incubated in a 50 µM monobromobimane in 100 µL PBS at 37 °C for 15 min. The fluorescence was quantified by spectrofluorimetry (VICTOR 2; PerkinElmer, Paris, France) with excitation wavelength of 380 nm and emission wavelength of 485 nm [34].

### 2.5. Detection of Reactive Oxygen Species

Cells were seeded at 1 × 10^4^ cells for NIH3T3 and HUVEC and 2 × 10^4^ for A549, MCF7, HT29 cells per well, in 100 µL complete medium, into 96-well plates and treated with garlic juice or allicin to obtain the final concentrations as indicated. The cells were incubated for 24 h at 37 °C and 5% CO_2_. At the end of the incubation period, cells were washed twice in PBS and incubated in 200 µM dichlorofluorescein-diacetate-solution for 15 min (Molecular Probes, Leiden, The Netherlands) and fluorescence was quantified by spectrofluorimetry (VICTOR 2; PerkinElmer, Paris, France) at excitation wavelength of 490 and 535 emission [36]. 

### 2.6. Estimation of Apoptotic Cells Using YO-PRO-1 Iodide

Cells were seeded at 1 × 10^4^ cells for NIH3T3 and HUVEC and 2 × 10^4^ for A549, MCF7, HT29 cells per well, in 100 µL complete medium, into 96-well plates and treated with garlic juice or allicin to obtain the final concentrations as indicated. The cells were incubated with allicin for 30 or 60 min, washed in PBS and subsequently incubated in the presence of 1 µM YO-PRO-1 iodide in 100 µL PBS (Molecular Probes, Eugene, OR, USA) for 20 min at room temperature. Fluorescence of YO-PRO-1 iodide is used as a measure for the relative quantity of apoptotic cells. Fluorescence was measured by spectrofluorimetry (VICTOR 2; PerkinElmer, Paris, France) at 490 nm excitation and 510 nm emission as described in [34].

### 2.7. Preparation of Garlic Juice and Quantification of Allicin by HPLC (High Performance Liquid Chromatography)

Garlic bulbs were purchased from the supermarket and stored at 4 °C in the dark until required. Axillary buds from the composite garlic bulb were peeled and weighed and a domestic juicer (Turmix Fabr. No. 1068; Turmix AG, Jona, Switzerland) was used to extract the juice. The juice was centrifuged at 3000× *g* for 10 min to separate the majority of the pulp from the liquid (Megafuge 1.0R; Heraeus Instruments, Osterode, Germany). Floating debris was scooped off the top of the liquid with a spatula and discarded. Filtering under pressure using a filter paper (MN615, Macherey-Nagel, Dueren, Germany) separated the remaining pulp from the pure extract (diaphragm vacuum pump; Vacuubrand GmbH, Wertheim, Germany). The filtrate was transferred to a sterile 50-mL Falcon tube. Allicin concentration was determined by HPLC. The method used was based on that of Krest and Keusgen [37]. Garlic juice was diluted 1:10 with HPLC-grade water and 1 mL was mixed with 1.5 mL of a 0.05 mg·mL^−1^ solution (in methanol) of butyl-4-hydroxybenzoate (internal standard). To protect the column, this mixture was first filtered using a 2 µm sterile-filter fitted to a syringe [38]. 

### 2.8. Allicin Synthesis

Allicin was synthesized by oxidation of diallyldisulfide with glacial acetic acid/H_2_O_2_ (per-acetic-acid) as previously described [39]. Diallyldisulfide (DADS, Sigma-Aldrich, Steinheim, Germany) was distilled under vacuum before being used and purity was checked with HPLC as described above. DADS (2 g = 13 mmol) was dissolved in 5 mL glacial acetic acid (Carl Roth, Karlsruhe, Germany) and 3 mL of ice-cold 30% hydrogen peroxide (Merck, Darmstadt, Germany) was added dropwise. The reaction proceeded for 30 min on ice and subsequently the temperature of the reaction mixture was allowed to increase to room temperature and to continue with stirring for two additional hours. The reaction was stopped by adding 25 mL deionized water (18.2 MΩ·cm^−1^) and was extracted twice with each 30 mL dichloromethane (Carl Roth, Karlsruhe, Germany). Acetic acid was removed by washing the extract several times with an aqueous 5% (*w*/*v*) NaHCO_3_ solution and was subsequently washed with distilled water until pH 6–7 was reached. The organic phase was evaporated *in vacuo* and the oil obtained was dissolved in 200 mL distilled water. The purity of the reaction product was checked by HPLC. If unreacted DADS was detected, an extraction with 0.1 vol % hexane was used to remove residues of DADS. Allicin was subsequently extracted with CH_2_Cl_2_, dried over anhydrous MgSO_4_ and concentrated *in vacuo*. Purity was checked by HPLC. 

## 3. Results

### 3.1. Effects of Allicin on Cell Viability

We compared the effect of both garlic juice, normalized to its allicin content and synthetic allicin on the cell viability using the MTT-assay. Viable cells reduce MTT to an insoluble violet coloured formazan which after solubilization in DMSO can be quantified spectrophotometrically at 550 nm. 

**Figure 1:**
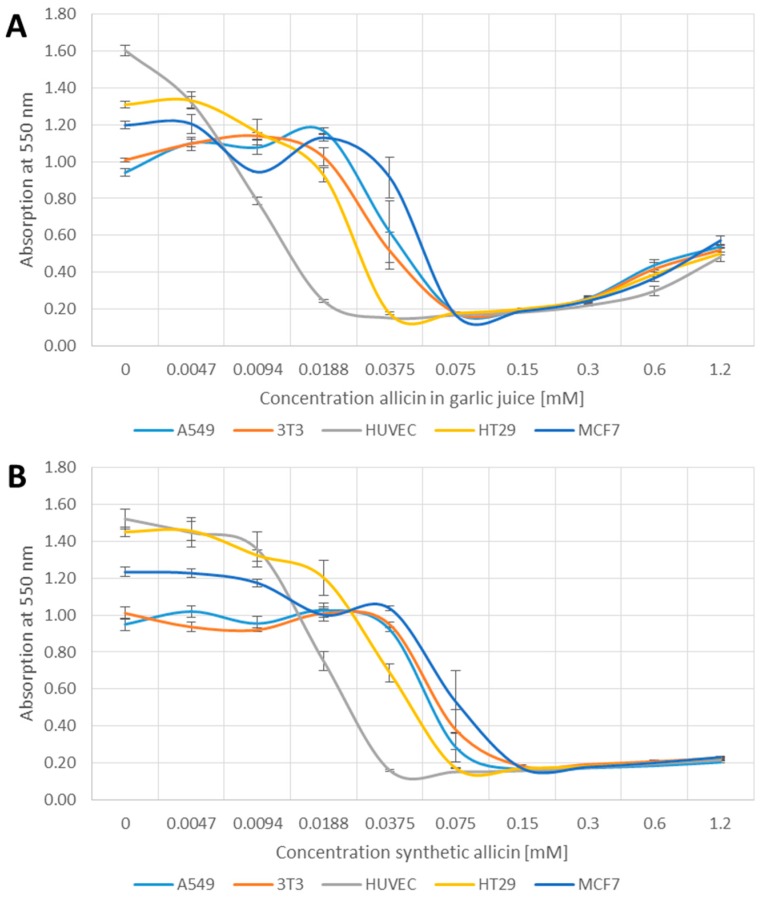
Effect of garlic juice normalized to its allicin concentration (**A**); and synthetic allicin (**B**) on the viability of different cell lines in the MTT (methylthiazoltetrazolium)-assay. All data points are means of three replicates; error bars show standard deviation.

Cell viability, in terms of MTT reduction, decreased in a concentration dependent manner for both garlic juice- and synthetic allicin-treated samples in an essentially similar manner (Figure 1). Of the cell lines tested, human endothelial vein cells (HUVEC) were most sensitive to allicin. HUVEC cell viability decreased by approximately 50% at 0.0094–0.0188 mM allicin (Figure 1A,B). There was a reduction in HUVEC cell viability at 0.0094–0.0188 mM allicin in garlic juice and 0.0188 mM synthetic allicin was significant compared to the other cell lines (*p* > 0.05, one-way-AnovaR, Holm–Sidak method). Human adenocarcinoma (A549), mouse fibroblast (3T3) and human mammary carcinoma (MCF7) cells were most resistant and show a decrease of 50% MTT absorption between 0.0375 and 0.075 mM allicin. These three cell types were significantly more resistant to 0.0375 mM allicin than the Human colorectal epithelial cells (HT29) (*p* > 0.05, one-way-AnovaR, Holm–Sidak method). HT29 cells were intermediate in sensitivity, showing approximately 50% reduction in viability at 0.188–0.375 mM allicin. This demonstrates that different cell-lines show intrinsically different sensitivity to allicin. The slight increase in A550 at higher concentrations of garlic juice (0.6 and 1.2 mM) was an artefact due to increasing turbidity of the solution and can be seen in all cell lines. The ranking of differential sensitivities of the cell lines to synthetic allicin or to allicin in garlic juice was the same i.e., HUVEC > HT29 > A549, 3T3 and MCF7 (Figure 1A,B).

### 3.2. Allicin Inhibits Cell Proliferation

**Figure 2:**
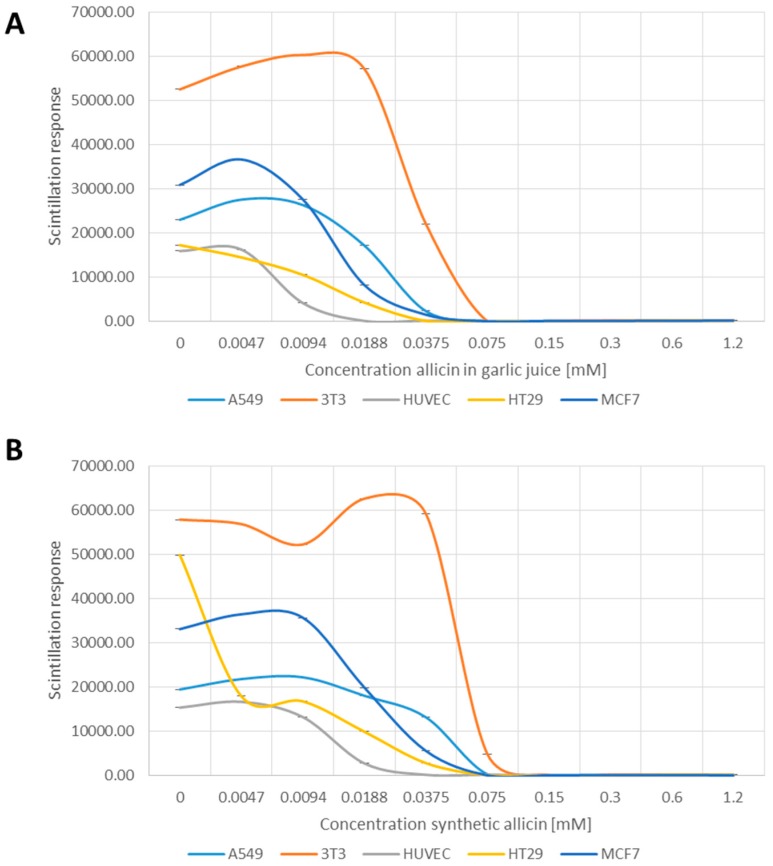
Effect of garlic juice normalized to its allicin concentration (**A**); and synthetic allicin (**B**) on cell proliferation as tested by incorporation of ^3^H labelled thymidine in DNA and measured by scintillation. All data points are means of three replicates; error bars show standard deviation.

The cell lines tested proliferated at different rates even in the untreated controls (Figure 2A,B). Nevertheless, allicin inhibited cell proliferation of all cell types in a concentration dependent manner, with the respective cell lines showing differential sensitivities. HUVEC cells were the most sensitive to allicin, with the incorporation of ^3^H-thymidine into newly synthesized DNA markedly reduced at 0.0094 mM allicin in garlic juice (*p* > 0.05, one-way-AnovaR, Holm–Sidak method) and completely inhibited at 0.0188–0.0375 mM allicin respectively (Figure 2A,B, *p* > 0.05, one-way-AnovaR, Holm–Sidak method). Mouse fibroblast 3T3 cells were the least sensitive, still showing normal ^3^H-thymidine incorporation at 0.0375 mM allicin where other cell lines were inactive (Figure 2A,B, *p* > 0.05, one-way-AnovaR, Holm–Sidak method). Human colorectal epithelial cells (HT29), human mammary carcinoma cells (MCF7) and lung epithelial carcinoma cells (A549) showed intermediate behaviour (Figure 2A,B). 

These data show that allicin is an inhibitor of cell viability and cell proliferation in a concentration dependent manner, but that different cell lines show different sensitivities. 

### 3.3. The Glutathione Pool Is Affected upon Treatment with Garlic Juice and Synthetic Allicin

We have shown previously that treatment of yeast cells with allicin leads to glutathione oxidation [39] and we therefore used monobromobimane as a fluorescent probe to measure glutathione in allicin-treated cells. Monobromobimane fluoresces upon binding reduced glutathione (GSH), whereas there is no reaction with oxidized glutathione (GSSG) or glutathione-adducts.

**Figure 3:**
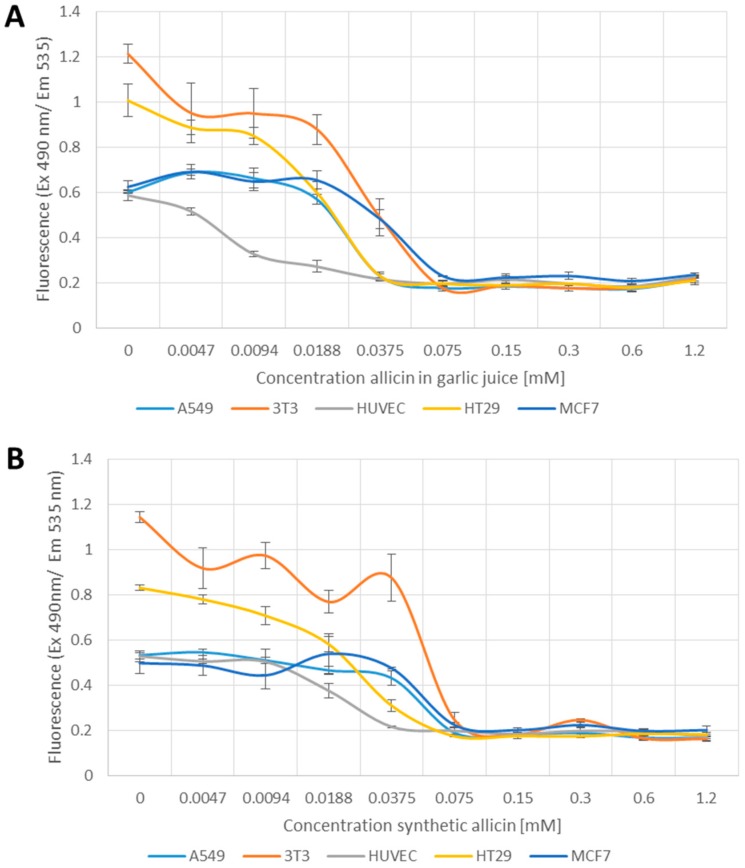
Measurement of reduced glutathione with monobromobimane. Different cell lines were pipetted into 96-well plates (3T3 (mouse fibroblast) and HUVEC (human umbilical vein endothelial cell) 1 × 10^4^ cells per well, A549 (human lung epithelium carcinoma), MCF7 (human breast cancer), HT29 (human colon carcinoma) 2 × 10^4^) and treated with allicin in garlic juice (A) or chemically synthesized allicin (B) for 24 h. Monobromobimane was added to a final concentration of 50 µM and incubated for 15 min before measurement. All data points are means of three replicates; error bars represent standard deviation.

Fluorescence corresponds to the quantity of reduced glutathione in the cells. The different cell lines used vary in the content of glutathione as shown in the untreated controls where it can be seen that A549, HUVEC and MCF7 cells have similar amounts of GSH and significantly less than 3T3 and HT29 cells (Figure 3A,B, *p* > 0.05 one-way-AnovaR, Holm–Sidak method). 

All cells showed a dose-dependent reduction in fluorescence in response to allicin treatment (Figure 3A,B) up to 0.0375 mM, showing that allicin leads either to an increased oxidation of the glutathione pool, or a reduction in its size, or both. All cell lines showed minimum fluorescence above 0.075 mM allicin with HUVEC cells being perhaps marginally more sensitive than the other cell lines and reaching minimum fluorescence at 0.0375 mM allicin (Figure 3A,B). The GSH level in the HUVEC cells already showed significant reduction compared to A549 and MCF7 cells after treatment with 0.094 mM allicin in garlic juice (Figure 3A, *p* > 0.05 one-way-AnovaR, Holm–Sidak method). In contrast, the 3T3 cell line showed significantly greater resistance than the other cell lines after treatment with 0.0375 mM allicin (Figure 3B, *p* > 0.05 one-way-AnovaR, Holm–Sidak method).

### 3.4. Allicin-Induced Accumulation of Reactive Species

Allicin oxidizes thiols and causes oxidative stress in its own right. However, the possibility that allicin causes the production of further reactive species, e.g., reactive oxygen species and organic radicals was investigated using 2′,7′-dichlorofluoresceine-diacetate (DCFDA). 

**Figure 4:**
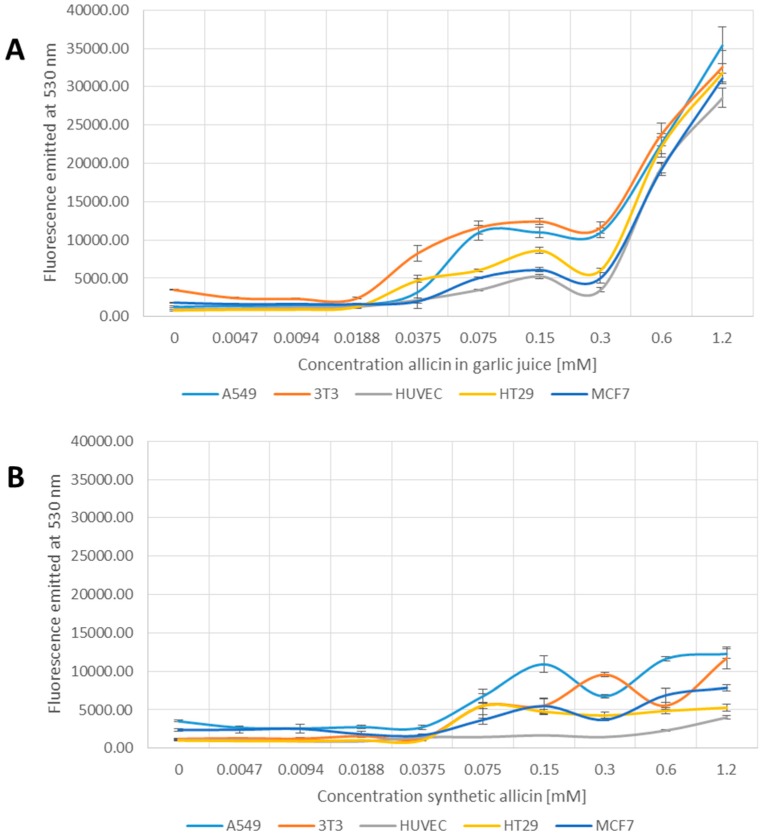
Detection of Reactive Species by 2′,7′-Dichlorofluoresceine-Diacetate (DCFDA) upon treatment with garlic juice (**A**); or synthetic allicin (**B**). Cells were incubated for 24 h with allicin, washed and treated with DCFDA in DMSO (dimethyl sulfoxide) to a final concentration of 50 µM for 15 min at room temperature, before fluorescence was measured at 530 nm. All data points are means of three replicates; error bars represent standard deviation.

In contrast to the tests reported so far, the 2′,7′-DCFDA assay for generation of reactive species showed a clear difference between garlic juice and synthetic allicin treatments (Figure 4A,B). In the samples treated with garlic juice, an increase in fluorescence occurred between 0.0188 and 0.375 mM allicin and there was a large jump in fluorescence at 0.3 and 0.6 mM allicin (Figure 4A). In contrast, in the samples treated with synthetic allicin, a slight increase in fluorescence was first observed only at a higher concentration (0.075 mM) and fluorescence levels remained low up to the highest concentration tested (1.2 mM allicin) (Figure 4B). This suggests that allicin, at lower concentrations, where effects were clearly seen in the MTT, ^3^H-thymidine and monochlorobimane tests, does not induce reactive species that can react with DCFDA. The slight increase in fluorescence seen at higher concentrations of synthetic allicin is possibly artifactual because the cells are very stressed at these concentrations (Figure 1, Figure 2 and Figure 3). The induction of DCFDA-fluorescence by low concentrations of garlic juice points to the presence of a substance other than allicin being responsible for this effect (Figure 4A,B). Interestingly, HUVEC cells showed no DCFDA-fluorescence even at the highest concentrations of synthetic allicin (Figure 4B).

### 3.5. Induction of Apoptosis by Allicin

**Figure 5:**
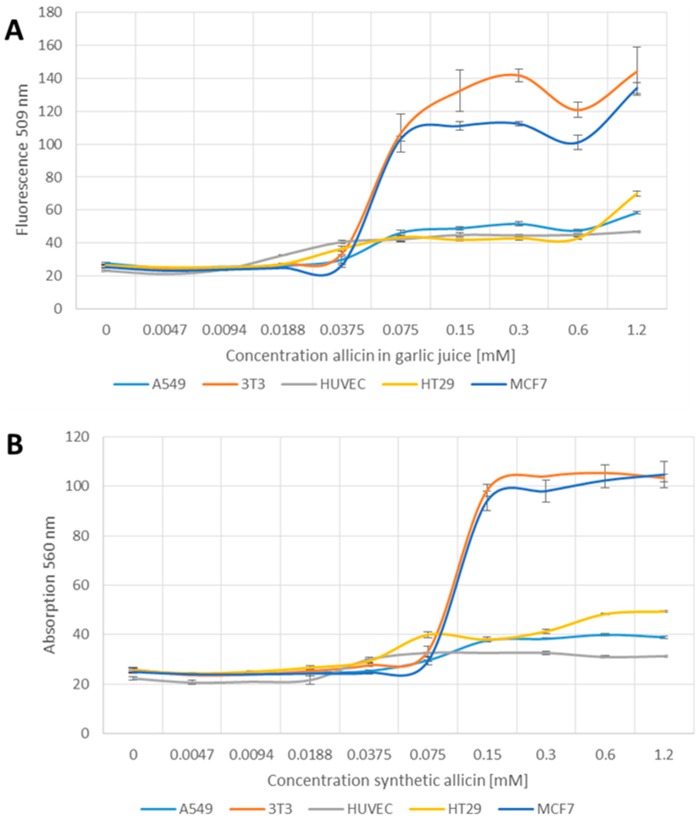
Induction of apoptosis by garlic juice normalized to its allicin concentration (**A**); and synthetic allicin (**B**). Different cell lines were seeded into 96-well plates (3T3 and HUVEC 1 × 10^4^ cells per well, A549, MCF7, HT29 2 × 10^4^) and treated with allicin in garlic juice or chemically synthesized for 1 h. After washing the cells, YO-PRO1 iodide was added to a final concentration of 1 µM and incubated for 20 min at room temperature. Relative fluorescence was measured at 509 nm after excitation at 490 nm. All data points are means of three replicates; error bars represent standard deviation.

YO-PRO-1 iodide is taken up by apoptotic cells but not by healthy cells. Allicin clearly induced apoptosis in the mouse fibroblast (3T3) and human mammary carcinoma (MCF7) cell lines above 0.075 mM (Figure 5A,B). In comparison, the other cell lines tested showed only a slight increase in fluorescence at 509 nm at allicin concentrations above 0.075 mM, up to the maximum concentration used (1.2 mM) (Figure 5A,B). The difference in apoptosis rates between the 3T3 and MCF7 cell lines compared to A549, HUVEC and HT29 cell lines were statistically significant at *p* > 0.05 (one-way-AnovaR, Holm–Sidak method).

To facilitate comparison between the effects of allicin on the different cell lines, the results of the tests are summarized together in Table 1.

## 4. Discussion

Since ancient times, garlic has been used worldwide for food and as a medicinal herb [1]. The health promoting properties of garlic have been shown in a plethora of studies (an overview is given in [40]), ranging from blood-lipid lowering effects [41] to antioxidant activity [42]. Allicin is the first and major volatile sulfur compound produced when garlic tissues are damaged and many of the beneficial claims made for garlic are ascribed to allicin. Allicin shows direct “antioxidant” activity by trapping radicals [26,43,44]. Nevertheless, allicin oxidizes thiol-groups, as has been shown experimentally in several studies [13,39]. These interesting properties and the fact that garlic is part of the human diet make it a very attractive subject for research to investigate the basis of health promotion and potential applications as a “nutraceutical” [6]. However, much research is performed with garlic preparations rather than pure substances e.g., with garlic oil or aged garlic extracts (AGE) [45]. Since the sulfur-compounds in freshly injured garlic tissue (especially thiosulfinates) are highly reactive, distilled or AGE preparations usually contain less thiosulfinates, but predominantly more polysulfanes and also ajoene or vinyl-dithiins, which also can have health promoting properties in their own right [41]. Furthermore, garlic juice contains many other (non-sulfur based) metabolites that show biological activity, such as phenolic compounds or saponins [46,47]. Thus, it is important to address the question as to whether a given biological activity can be correlated with the allicin content of garlic juice, or whether it might be due to other substances present. 

Although allicin has beneficial health effects, it is also a strong biocidal agent with antimicrobial efficacy comparable to conventional antibiotics [6,48]. Allicin is also toxic to mammalian cell lines, and anti-tumour activity has been documented [28,49]. We tested the comparative effects of synthetic allicin and garlic juice normalized to the allicin content on a variety of mammalian cell lines in order to address some of the questions outlined above.

### 4.1. Synthetic Allicin vs. Allicin in Garlic Juice

With the exception of the DCFDA test for reactive species, all other assays showed very similar concentration-dependent trends for the effects of synthetic allicin and allicin in garlic juice on any given cell line (Figure 1, Figure 2, Figure 3, Figure 4 and Figure 5, respectively). Thus, it can be tentatively concluded that the effects of raw garlic juice on the tested cell lines is largely due to the allicin content. This is an important observation, given that garlic is an important culinary item, often consumed raw in some dishes and salads, e.g., tzatziki. 

In the case of DCFDA, the increasing induction of fluorescence by low concentrations of garlic juice above 0.0375 mM allicin and minor effect by synthetic allicin points to the presence of a substance or substances other than allicin being responsible for this effect of diluted crude garlic juice (Figure 4A,B). Thus, it seems that allicin did not induce any DCFDA-detectable reactive species up to 0.075 mM and therefore that the effects seen in the other assays at lower concentrations are due to allicin itself and not secondary effects from reactive species produced as a result of allicin treatment.

### 4.2. Effects of Allicin on Cell Viability and Proliferation

The MTT assay is based on the reduction of a tetrazolium-salt by mitochondrial reductases such as succinate dehydrogenase. Although commonly used for determination of cell viability, the MTT-assay can have drawbacks for the evaluation of the toxicity of a given test substance. The MTT assay is based on the presumption that respiratory activity coincidences with cell-viability. Allicin is able to inhibit many cysteine-containing enzymes [15] and if some mitochondrial reductases were targets of allicin, inhibition might lead to less MTT-reduction, although the cells are still viable. Alternative viability assays based on membrane-permeability (e.g., propidium-iodide) are also problematic since allicin is membrane active and forms transient pores in membranes [38]. Nevertheless, allicin treatment decreased MTT-reduction in all tested cell lines in a dose-dependent manner and, given the proviso outlined above, may be taken to indicate that allicin reduced cell viability [50]. High concentrations of garlic juice (>0.3 mM allicin) led to an apparent slight recovery in MTT-absorption. Similar to the effect of garlic juice on DCFDA-fluorescence, this seems to be an artefact. A possible explanation could be the induction of reactive species by garlic juice (Figure 4A). Thus, for example, strongly reducing superoxide radicals would lead to a reduction of MTT. We have observed a similar effect in yeast cells treated with menadione (vitamin K), a strong superoxide-generator. Even at menadione concentrations that were clearly toxic to the yeast cells, MTT absorbance increased and was correlated with the enhanced production of superoxide, measured by a lucigenin-assay. HUVEC cells were the most sensitive to allicin 

The effect of a test substance on cell proliferation rate, in addition to its effect on cell viability, is also a good indicator of toxicity. Allicin has been reported in a number of studies to inhibit cell proliferation [27,47,51]. Allicin targets tubulin, which forms the mitotic spindle, and thus prevents cell division [18]. Our results confirm these earlier observations and show that allicin reduced the incorporation of ^3^H-thymidine into DNA in a dose-dependent manner. The sensitivity of cell proliferation in the different cell lines to allicin varied. The 3T3 fibroblasts divided faster than the other cell lines in the untreated control and were most resistant to allicin. HUVEC cells were the most sensitive to allicin (Figure 2A,B).

### 4.3. Effect of Allicin on the Glutathione Pool

Allicin is a thiol-reagent and reacts easily with glutathione, forming S-allylmercaptoglutathione (GSSA) and leading to an increased production of GSSG [13,39,52]. The state of the cellular GSH-GSSG redox couple is closely linked to the physiological status cells [22,53], and we previously showed the correlation between allicin-induced glutathione oxidation and induction of apoptosis in *Saccharomyces* [39]. Therefore, we assayed changes in the amount of reduced glutathione in the various cell lines with monobromobimane, which reacts with the free thiol group forming a fluorescent adduct, most likely in a glutathione-S-transferase dependent manner [54]. Lower fluorescence in comparison to the untreated controls can be interpreted as oxidation of glutathione to GSSG or depletion of the GSH glutathione pool (e.g., by formation of a mixed-disulfide). A dose-dependent decrease of fluorescence was observed in all cell lines, suggesting that allicin reacts with and depletes the GSH pool (Figure 3A,B). Interestingly, HUVEC cells, which were most sensitive to allicin in the MTT-assay, showed a very low initial GSH content and the cells were quickly depleted of GSH by allicin treatment.

In order to test whether allicin might be inducing oxidizing conditions in the cells which lead to a production of oxidizing reactive species, we incubated the cell lines with 2,7-DCFDA. Although DCFDA is sometimes reported as a specific indicator for Reactive Oxygen Species (ROS) [55,56], it becomes apparent that 2′,7′-DCFDA is a fairly non-specific and indirect detector of several Reactive Species [56]. Thus, we prefer to describe our results as a measurement of Reactive Species in general rather than ROS-production in particular. Whereas synthetic allicin caused only a slight DCFDA fluorescence at high concentrations, in contrast, garlic juice triggered massive formation of Reactive Species. We also tested the direct reaction of either garlic juice or synthetic allicin with 2′,7′-DCFDA and found no increase in fluorescence, suggesting that the observed RS accumulation is indeed induced in the cells, not due to a direct reaction with the fluorescent dye. Since garlic juice is, in contrast to synthetic allicin, a complex mixture of different compounds, it is likely that the massive RS-induction by garlic juice is due to the presence of other compounds. Possibly, glycosylated proteins in the garlic juice (e.g., the alliinase enzyme) are acting as “elicitors” of a kind of “oxidative burst”. Immune cells react sensitively to many glycosylated proteins in this way [57]. Although macrophages are associated with oxidative burst, other cell types respond to specific stimuli with the production of Reactive (oxygen) species [58,59]. However, synthetic allicin did not influence the oxidative burst in macrophages [60]. The very different response of the cell lines to synthetic allicin and garlic juice in causing changes of DCFDA fluorescence clearly illustrates that caution is always necessary when interpreting the effects of “complex preparations” from nature, even if they contain known, physiologically active molecules. Many studies set an “allicin” equivalent with directly extracted garlic, or complex mixtures containing allicin with different degrees of purity [61,62]. Our data clearly show that it is crucial to clearly define the source and purity of the allicin used. 

### 4.4. Effect of Allicin on Inducing Apoptosis

Since allicin is a redox-toxin [39] and the cellular redox-status is closely linked to the physiological status of cells [53], we expected that allicin might be able to induce apoptosis by a redox-mechanism, as seen before in the model fungus *Saccharomyces cerevisiae* [39]. Therefore, we tested for apoptotic cells by using the YO-PRO-1 iodide fluorescent dye that can enter apoptotic cells but not non-apoptotic cells [63]. Interestingly, there were pronounced differences between rates of allicin-induced apoptosis between the various cell types. While 3T3 and MCF7 cells showed a dramatic increase in YOPRO-1 signal, indicating a high rate of apoptotic cells, the other cell types only exhibited a very slight increase, even up to the highest allicin concentrations where the MTT and ^3^H-thymidine tests indicated that cells were probably dead. This suggests that while 3T3 and MCF7 cells die apoptotically in response to allicin, the HUVEC, A549 and HT29 cells undergo non-apoptotic cell death, i.e., necrosis. However, as already stated, the induction of apoptosis by allicin has been reported for numerous cell types including SiHa cells (human cervical cancer cell line), L-929 (murine fibrosarcoma), SW480 (human colon cancer) and HeLa (human cervical cancer), AGS (a human gastric carcinoma cell line), HT-9/HL-60 (colon adenocarcinoma and MDR1 hyperexpressor) and HCT-116, LS174T, HT-29 and Caco-2 (human colon cancers) [16,17,20,24]. The mechanisms leading to the allicin-induced non-apoptotic cell death of HUVEC, A549 and HT29 cells merit further investigation. Interestingly, the pattern of apoptosis induction does not correlate with the size or behaviour of the cellular glutathione pool for the various cell types. Thus, HUVEC cells showed a low rate of apoptosis but had a small GSH pool which was rapidly oxidized, whereas 3T3 cells showed a high apoptotic rate and had a large GSH pool which in theory should protect them better from the allicin oxidative insult (Figure 3A,B). Furthermore, A549, MCF7 and HUVEC cells all start out with similar GSH pools but show different dose-dependent patterns of GSH oxidation after allicin treatment (Figure 3A,B). The activity of glutathione reductase and the availability of NADPH (reduced nicotinamide adenine dinucleotide phosphate) as a co-substrate to reduce GSSG back to GSH may be factors that differ between the various cell lines. In cells where the redox-buffering is weak, maybe the redox in the cells is pushed rapidly into a range where the cells no longer have the metabolic integrity to execute the cell death program associated with apoptosis, and the cells simply necrose.

## 5. Conclusions

Allicin is part of the human diet. Because of the purported beneficial health effects of allicin, it is important to catalogue its effects on different cell types and separate allicin-specific effects from effects due to other garlic constituents. The work presented here contributes to that knowledge base.

## Figures and Tables

**Table 1:** A comparison between the various cell lines tested in their relative responses to allicin. + = low, ++ = intermediate, +++ = strong, − = no response.

Test	A549	3T3	HUVEC	HT29	MCF7
MTT (viability)	+	+	+++	++	+
^3^H-thymidine (proliferation)	++	+	+++	++	++
Monobromobimane (GSH)	++	+	+++	++	++
DCFDA (reactive species)	−	−	−	−	−
YO-PRO-1 (apoptosis)	+	+++	+	+	+++
